# An ethnobotanical survey on hormozgan province, Iran

**Published:** 2013

**Authors:** Omid Safa, Mohammad Amin Soltanipoor, Soheil Rastegar, Mahnaz Kazemi, Khadijeh Nourbakhsh Dehkordi, Alireza Ghannadi

**Affiliations:** 1 *Bandar Abbas School of Medicine, Hormozgan University of Medical Sciences, Bandar Abbas, I. R. Iran*; 2 *Hormozgan Agricultural and Natural Resource Research Center, Bandar Abbas, I. R. Iran*; 3 *School of Pharmacy, Isfahan University of Medical Sciences, Isfahan, I. R. Iran*; 4 *District One Education Department, Isfahan Education Administration, Isfahan, I. R. Iran*; 5 *Pharmaceutical Sciences Research Center, Isfahan University of Medical Sciences, Isfahan, I. R. Iran*

**Keywords:** Ethnobotany, Hormozgan, Iran, Medicinal Plants, Persian Gulf

## Abstract

**Objective:** The present study is based on an ethnobotanical research project conducted in Hormozgan province that is located in south of Iran, bordering waters of the Persian Gulf and Oman Sea. This survey was carried out in order to recover the ethnobotanical and ethnomedicinal knowledge of the residents of this province. They are using medicinal and functional plants for treating or preventing several diseases.

**Materials and Methods: **Ethnobotanical data sheets were run with the native inhabitants and people of the province by arranging frequent field trips to different parts of the province and direct interviews with them especially those who were more familiar with the plants and their usage**.**

**Results: **A total of 150 plant species belonging to 53 families were recorded for their ethnobotanical and ethnomedicinal uses among the people of province. The records were developed by scientific names, family names, local names, medicinal parts used, different ways of their application, and traditional uses of the plants. There was high compliance in the use of plants in painful disorders, gastrointestinal, and dermatological diseases.

**Conclusion: **This study revealed that the people of Hormozgan province have a rich knowledge of natural resources. The use and consumption of medicinal plants are still important parts of their life. Rational use of native medicinal plants may benefit and improve their living standards and quality of life. The results of this study can be used as a basis for selecting herbs for further pharmacological, phytochemical, and pharmacognostical studies.

## Introduction

Ethnobotany, understanding of knowledge systems through using anthropological methods, and ethnomedicine, as its branch are as old as man himself. Ethnobotany and ethnomedicine consider the collection of useful medicinal plants by a group of people and describing different uses of them. Utilizing plants for medicinal purposes has been done since the dawn of man (Namsa et al., 2011 ▶; Oliveira et al., 2011 ▶). Little by little people observed special interesting effects from each plant. Some of these people became experts in treating several ailments and illnesses using efficient plants and then they passed their knowledge to others verbally or by personal experiences (Kunwar et al., 2010 ▶; Zolfaghari et al., 2012 ▶). During this processes some information may be lost, vanished, or forgotten due to the society modernization so in this study we decided to collect these valuable documents and traditional knowledge in one of the southern provinces of Iran, Hormozgan. As a result, we can improve the quality of life and living standards of the native people by rational and standard using of medicinal plants along with effective synthetic drugs (Namsa et al., 2011 ▶; Oliveira et al., 2011 ▶). 

Nowadays, almost 80% of world population uses medicinal plants for their primary healthcare needs because they are effective, cheap, and available (WHO, 2007 ▶). About 70,000 plant species are used in traditional medicine and nearly a tenth part of them are used in Asia. Iran which is located in southwest Asia, in the northern hemisphere, contains rich ecosystems and biodiversity due to the various climatic conditions and geographical characteristics (Bhattarai et al., 2010 ▶; Mirdeilami et al., 2011 ▶; Naghibi et al., 2005 ▶). Iran is surrounded by three seas and a passage toward the oceans. The flora of the country contains more than 8000 species and several of them are used in traditional Iranian medicine (Ghahreman, 1973 ▶; Namsa et al., 2011 ▶; Sabzian, 2008 ▶). A few ethnobotanical researches have been done in Iran and there is no previous published records on ethnobotanical knowledge from the Hormozgan province (Amin, 1991 ▶; Ghassemi Dehkordi et al., 2012 ▶; Gholassi Mood, 2008 ▶; Ghorbani, 2005 ▶; Ghorbani et al., 2006 ▶; Mazandarani, 2006 ▶; Miraldi et al., 2001 ▶; Mirdeilami et al., 2011 ▶; Mosaddegh et al., 2012 ▶; Naghibi et al., 2005 ▶; Shams Ardekani et al., 2011 ▶; Sharififar et al., 2010 ▶; Shokri and Safaian, 1993 ▶; Soltanipoor, 2005 ▶; Zolfaghari et al., 2012 ▶). 

**Geographical and historical overviews**

**Figure 1:**
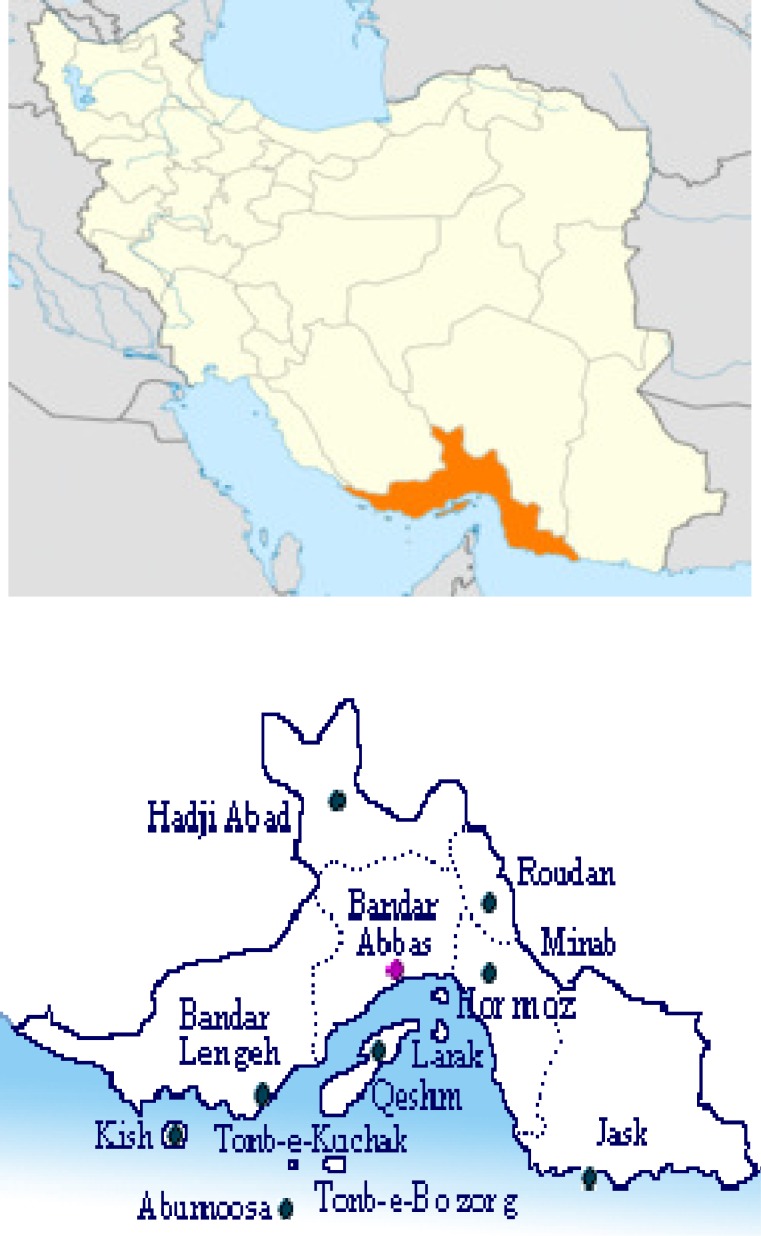
Map of the study area (Hormozgan provice, south of Iran, bordering waters of the Persian Gulf and Oman Sea) (Zaeifi, 2001; Sabzian, 2008).

Hormozgan province district is situated in the southeast of Iran (Figure 1). More than 70% of the province is covered by mountains and hills thus it is a mountainous region (IGA, 1983 ▶; Zaeifi, 2001 ▶). The district is bounded by Kerman province in the north and northeast, Fars and Bushehr provinces in the west and northwest and Sistan and Baluchestan province in the east. The southern parts of this province which is surrounded by warm waters of the Persian Gulf and Oman Sea is approximately 900 km. This province is located between northern latitude 25⁰ 24' to 28⁰ 57' and eastern longitude 53⁰ 41' to 59⁰ 15'. It occupies an area of 70697 km^2^ (IGA, 1983 ▶; Sabzian, 2008 ▶; Mozaffarian, 1991 ▶; Soltanipoor, 2005 ▶; Zaeifi, 2001 ▶).

The history of Hormozgan province is mixed with the history and geography of the Persian Gulf. Hormoz straight, one of the today’s most sensitive and vital waterways, is situated in political territory of this province. Bandar-Abbas, Bandar Lengeh, Minab, Bandar Charak, Bandar Jask, Roudan, Khamir, Parsian, Sirik, Hadji-Abad, Kish, Hormoz, Abu-Moosa, and Gheshm islands constitute the famous townships and areas of the province. Bandar Abbas is the capital of Hormozgan province and Gheshm is the largest island of the Persian Gulf (Soltanipoor, 2006 ▶; Sabzian, 2008 ▶; Attar et al., 2004 ▶; Shahi et al., 2011 ▶).

**Climate and vegetation types**

Three types of climate exist in this province. The natural vegetation is forest, rangeland, and desert. Relatively high humidity, irregular, and little rainfalls with hot weather result in growing some special and native plants. Several of these plants are being used for medical purposes by indigenous people (IGA, 1983 ▶; Soltanipoor, 2005 ▶; Zaeifi, 2001 ▶). The average temperature affected by humidity is moderate and rarely gets higher than 45 ⁰C in summers. In the deserts, the temperature is about 0 ⁰C but there is no frigid weather in winters. The annual rainfall is less than 250 mm and relative humidity is more than 80% (IGA, 1983 ▶; Morid et al., 2001 ▶; Sabzian, 2008 ▶; Zaeifi, 2001 ▶). 

There are 900 plant species in the province that too many of them are medicinal. Different climate conditions result in growing of specific plants such as special marine plants and mangrove (*Avicennia marina*) forests which are very rare (Mozaffarian, 1991 ▶; Soltanipoor, 2005 ▶; Zaeifi, 2001 ▶). Iranian Mangrove forests as unique and highly productive ecosystems of the world were recorded in the Persian Gulf and Oman Sea by Eratosthenes, who was a great geographer about 2300 years ago. Iran has the highest acreage of natural mangrove forest (Ghasemi et al., 2010 ▶; Sabzian, 2008 ▶).

## Materials and Methods

Data collection and field trips were arranged in order to collect information about traditional and folk knowledge of medicinal plants by the local inhabitants, native practitioners, and old people for the treatment or prevention of several ailments. Direct interviews with local people especially those who were more familiar with the herbs and their usage, were the main method. Ethnobotanical data sheets were used to document the medicinal knowledge by holding the direct interviews with people and gathered information was checked again with the people of other neighboring areas (Bhattarai et al., 2010 ▶; Mosaddegh et al., 2012 ▶; Sharififar et al., 2010 ▶; Zolfaghari et al., 2012 ▶).

All collected plant specimens were dried, pressed and authenticated with the help of available literature and flora (Ghahreman, 1973 ▶; Rechinger, 1982 ▶). After the scientific name identification of the plants, the specimens were deposited in the herbarium of Hormozgan Agricultural and Natural Resource Research Center, Bandar Abbas. The popular names of plants as well as their pronunciations were recorded.

## Results

In this paper information of 150 medicinal plant species were collected. This information contains scientific names, family names, local names, medicinal parts used, ways of their application, and traditional uses of them. A part of therapeutic properties of the mentioned plants were found in scientific resources and literature. This could be valuable since people use these plants because of their local useful effects. The information was sorted in Table 1 alphabetically.

**Table:** 

**No.**	**Scientific name**	**Family name**	**Local or official name**	**Part(s) used**	**Ways of application**	**Uses/ Ailments treated**
**1.**	*Abutilon fruticosum* Guill. & Perr.	Malvaceae	Garshem	Flower, leaves, seed	Decoction fresh organ	Wound, acne, pustule, cold, emollient, bronchitis
**2. **	*Abutilon hirtum* (Lam.) Sweet	Malvaceae	Sherbejan	Flower, leaves, seed	Decoction fresh organ	Cold, bronchitis, emollient, acne, wound, pustule, wound healing
**3. **	*Abutilon muticum* (Delile ex DC.) Sweet	Malvaceae	Kharmalchook	Seed, flower, leaves	Decoction fresh organ	Wound healing, acne, pustule, cold, bronchitis, emollient
**4.**	*Acanthophyllum bracteatum* Boiss.	Caryophyllaceae	Chubake bargdar	Root	Brew poultice powder	Decongestant, diuretic, wound healing, joint pain, sciatica, emmenagogue
**5.**	*Acanthophyllum squarrosum* Boiss.	Caryophyllaceae	Chubakeriz	Root	Brew poultice powder	Cold, joint pain, sciatica, gluteus pain, wound healing, diuretic, kidney stones
**6.**	*Achillea eriophora* DC.	Compositae	Benjerashk, berenjasf, sarzatdu	Flower, leaves	Poultice powder	Antipyretic, insects bite, bee bite, snake bite, scorpions bite, wound healing, bleeding
**7.**	*Achillea wilhelmsii* C. Koch	Compositae	Gol sarbarze, Sarbarze, sarbarde, sarzard	Aerial parts esp. flower	Decoction powder	Diarrhea, stomachache, fever, bile, anti-parasite, snake bite, scorpions bite, muscle tonic, fatigue in newly delivered ladies, headache, cold
**8.**	*Aerva persica* Merr.	Amaranthaceae	Porzu, ethag, gormenaku	Leaves	Powder	Wound
**9.**	*Alyssum homalocarpum* Boiss.	Cruciferae	Ghodume	Seed	decoction	Intestine emollient
**10.**	*Ammi majus* L.	Umbelliferea	-	Seed	Brew powder	Flatulency, diuretic, carminative, tonic, digestant, dyspepsia
**11.**	*Amygdalus lycoides* Spach	Rosaceae	Kutur, kulem	Aerial parts	Decoction fresh organ	Headache, burning wounds
**12.**	*Amygdalus scoparia* Spach	Rosaceae	Badam talkh	Twigs, resin	Decoction fresh organ	Pain of different parts, back pain, foot pain, pertussis
**13.**	*Anagalis arvensis* L.	Primulaceae	Gol nili, naem, golbadami, mash koshu, chehm kagu	Aerial parts	Brew poultice decoction	Nephritis, insect bites, jaundices, diuretic, painful wounds, bile wound healing, expectorant, chest & urination disease
**14.**	*Anchusa italica* Retz.	Boraginaceae	Gavzaban, gavzabane koohi	Leaves	Decoction	Cold, sore throat, chest pain
**15.**	*Andrachne aspera* Spreng.	Euphorbiaceae	Darmamaron	Stem	Fresh organ	Pterygium
**16. **	*Artemisia Aucheri* Boiss.	Compositae	Deraym koohi	Leaves	Fresh organ powder	Stomachache
**17.**	*Artemisia scoparia* Waldst. & Kit	Compositae	Salbaku, muleng, omeabid	Leaves	Decoction fresh organ	Flatulency in children, joint pain & rheumatism, hydrocele
**18.**	*Arundo Donax* L.	Gramineae	Ghamish	Root rhisome	Decoction	Alopecia , diuretic
**19.**	*Astragalus fasciculifolius*	Papilionaceae	Gonza, chetat, genjar	Resin	Powder	Cold, fatigue, tightening bone fractures
**20.**	*Astragalus mucronifolius* Boiss.	Papilionaceae	Genjarekhari	Root	Decoction	Back pain, bone fracture
**21.**	*Avicennia marina* (Forssk.) Vierh.	Avicenniaceae	Harra	Fruit Root resin	Poultice fresh organ	Snakebite, contraception, sexual stimulant, sexual enhancing, abscess, blotch & wound
**22.**	*Bienteria cycloptera* Bunge	Chenopodiaceae	Andere, samsil, samsul	Leaves	Decoction	Hyperlipidemia, hyperglycemia
**23.**	*Blepharis persica* (Burm.) O. Kuntze	Acanthaceae	Joojadoo, kisedokhtan	Leaves, seed, root	Fresh organ	Appetizing, astringent, energizer, tonic, mental discomforts, diuretic, styptic, anti-inflammatory, antitussive, hepatic and splenic discomforts
**24.**	*Boerhavia diffusa* L.	Nyctaginaceae	Shabrangieafshan	Leaves, root	Fresh organ	Joint pain, appetizing, tonic, expectorant, carminative, diuretic, jaundice, internal inflammation, edema
**25.**	*Brassica Tournefortii* Gouan	Cruciferacea	Kalam	Aerial parts	Fresh organ	Food additive, appetizing
**26.**	*Bunium persicum* (Boiss.)	Umbelliferae	Zireh	Seed	Decoction powder	Toxicity, antitussive, decongestant, children earache, newly delivered ladies recovery
**27.**	*Capparis cartilaginea* Decne	Capparidaceae	Kavarzeh barg pahn, naloostak	Leaves, fruit	Fresh organ	Rheumatism, joint pain, wounds
**28.**	*Capparis deciduas* (Forssk.) Edgew.	Capparidaceae	Kalir	Leaves, twig	Powder	Antipyretic
**29.**	*Capparis spinosa* L.	Capparidaceae	Konar eshkal, karaveng, kavarzah	Leaves, fruit	Fresh organ	Joint pain, rheumatism, abdominal pain
**30.**	*Capsella bursa-pastoris* (L.)	Cruciferae	Looseroo, loosiroo	Leaves, stem, latex	Poultice fresh organ	Bleeding, superficial inflammations, wound healing
**31.**	*Caralluma edulis* Benth.	Asclepiadaceae	Doghabis, howraghu	Stem, succulent stem	Fresh organ	Parasitic diseases, used as vegetable
**32.**	*Caralluma oxyacantha *	Compositae	Karajusk, hesk, karala, kharkala	Leaves	Decoction	Kidney pain
**33.**	*Caralluma tuberculata*	Asclepiadaceae	Moghmaar, maarangoosh	Succulent stem	Fresh organ	Parasite repellent
**34.**	*Cassia italica* (Miller) F.W. Andrews	Caesalpinaceae	Setaap,hashi, kowsen,kowchen, gush ahu , setaag,	Leaves	Powder	Laxative, cathartic
**35.**	*Centaurea Bruguierana *(DC.)	Compositae	Balehbord, badavard, badavardeh, kharkharangoo	Leaves, flower	Decoction	Headache, antipyretic, anti-scorpions bite
**36.**	*Centaurium tenuifolium* (Hoffm. & Link) Fritsch	Gentianaceae	Ghontorion	Flower, leaves	Brew fresh organ	Wound healing, hepatic and nephritic distress, jaundice, young girls anemia, diabetes, eczema
**37.**	*Cleome brachycarpa* Vahl. ex DC.	Capparidaceae	Glepar, ruzgardesh	Aerial parts esp. leaves & stems	Decoction powder	Toxicity of snake bite & scorpions bite
**38.**	*Cocculus pendulus *(J.R. & G. Forst.) Diels	Menispermaceae	Zamoor, zamer	Root	Decoction	Antipyretic
**39.**	*Convolvulus glomeratus* Choisy	Convolvulaceae	Pichak	All parts	Fresh organ	Cathartic
**40.**	*Convolvulus leptocladus *Boiss.	Convolvulaceae	Rontazg	Root	Powder	Cathartic
**41.**	*Convolvulus spinosus* Burm.	Convolvulaceae	Pichak khari	Flower	Fresh organ	Cathartic, antiparasite
**42.**	*Conyza Canadensis* (L.) Cronq.	Compositae	Pirbaharakebagh	Whole plant esp. leaves	Brew	Wound healing, kidney stones, bleeding during menstruation, elimination of female secretions
**43.**	*Corchorus tricularis*	Tiliaceae	Katan	All aerial parts	Decoction fresh organ	Emollient
**44.**	*Cornulaca monocantha*	Chenopodiaceae	Javen, sekhar, kharune	Leaves	Fresh organ	Snake bite, scorpion bite, bee bite, wound healing
**45.**	*Cotoneaster kotschyi* Klotz	Rosaceae	Shirkhesht	Fruit	Decoction	Jaundice, cooling
**46.**	*Cymbopogon Olivieri* (Boiss.) Bor	Gramineae	Paashaam, nagerd, zeghbar, maade	Green leaves	Decoction	Cooling, stomachache, bone pain, fever lowering, measles, cold
**47.**	*Cyperus rotundus* L.	Cyperaceae	Pizg	Rhizome, root	Decoction fresh organ	Dysentery, diuretic, gastric ailments, diarrhea, menstruation inducer, sweating inducer, parasite repellent, wound healing, pruritus, appetizing
**48.**	*Dalbergia sisso* Roxb.	Papilionaceae	Jak	Stem bark	Decoction	Tonic, appetizing, abortion, emollient, indigestion, dysentery, antiparasite
**49.**	*Daphne oleoides *Schreb.	Thymelaeaceae	Terbid, terbit	Peel, stem, leaf	Decoction fresh organ	Antipyretic, elimination of the pruritus & pain of a kind of insect bite called sisko
**50.**	*Datura innoxia *Miller	Solanaceae	Megena, permengenas, kopakemengenas	Leaves, flower, seed	Decoction brew	Demulcent in asthmatic patients, cutaneous disease, washing swelling feet, antitussive
**51.**	*Demostachia bippinata L.*	Graminae	Kertaki, kertah	Root	Fresh organ	Jaundice, peptic discomforts, emesis, nephritic disease, rash, kidney stones
**52.**	*Dionysia revolute*a Boiss.	Primulaceae	Esfande mohammadi, gazir, gurzi	Aerial parts	Decoction Fresh organ powder	Antiseptic, wound healing, gastric distress, stomachache, joint pain, insect bite, emollient in cold, ecchymosis, fatigue
**53.**	*Dodonaea viscose* (L.) Jacq.	Sapindaceae	Shahaf, mordang, nader, naterak	Leaves	Fecoction fresh organ	Headache, bone pain, foot pain, papule & blotch healer
**54.**	*Echinops Aucheri* Boiss.	Compositae	Shekar kooh	Resin	Decoction	Emollient in cold & pectoralgia, laxative
**55.**	*Eclipta prostrate *(L.) L.	Compositae	Masture khabideh	Aerial parts	Decoction fresh organ	blood purifier
**56.**	*Ephedra major* Host	Ephedraceae	Houm	Stem, root, fruit	Decoction	Rheumatism, syphilis, respiratory ailments
**57.**	*Erodium cicutarium *(L.) L’Her.	Geraniaceae	Sikh shabgard	Root	Decoction	Toothache
**58.**	*Euphorbia larica* Boiss.	Euphorbaceae	Paah, paragh	Latex	Fresh organ	Wound healing
**59.**	*Euphorbia osyridea* Boiss.	Euphorbiaceae	Rutazgh	Root	Fresh organ	Constipation
**60.**	*Euphorbia turcomanica* Boiss.	Euphorbiaceae	-	Leaves	Fresh organ	Cold
**61.**	*Ferula assa-feotida* L.	Umbelliferae	Heng, anghuzeh, angosht gande,engez	Resin or latex, root	Powder	Insect repellent, wound healing, ear ache, antiseptic, parasite repellent
**62.**	*Fortuynia bungei* Boiss.	Cruciferae	-	Seed, twig	Fresh organ	Bone pain, joint aches, flatuosity
**63.**	*Francoeuria undulate *(L.) Lack	Compositae	Porz, tahre	Leaves	Fresh organ	Children complaints
**64.**	*Fumaria parviflora *Lam.	Fumariaceae	Shahtareh, shatareh	Leaves, stem	Decoction	Pain relief, back cramps, infected wound, skin disease, stomachache
**65.**	*Gailonia Aucheri * Jaub. Spach	Rubiaceae	Toosoo, boogandoo, titisko, khargol, kartos	Leaves, twig, flower	Fresh organ brew	Toothache, rheumatism, flatuosity, diarrhea, gonalgia, gnathitis
**66.**	*Geranium rotundifolium* L.	Geraniaceae	Suzanuk	Root	Fresh organ	Diarrhea, diuretic, astringent
**67.**	*Gisekia pharnaceoides* L.	Molluginaceae	-	All parts	Fresh organ Powder	Digestant, anti-parasite, wound healing, appetizing, bronchitis, cutaneous discomforts, edema of nose mucous, anti parasite
**68.**	*Glaucium flavum* Crantz	Papaveraceae	Shaghayegh shakhdarezard	seed	Powder	Laxative
**69.**	*Glossonema variance* Decne	Asclepiadaceae	-	Fruit	Fresh organ	Cooling, digestant
**70.**	*Glycyrrhiza glabra* L.	Papilioceae	Shirinbayan, choobshirin, mahak, marah	Leaves, stem, root	Fresh organ decoction	Joint pain, measles, gastric ulcer, duodenum ulcer, cold
**71.**	*Grantia Aucheri* Boiss.	Compositae	Halamook, halamoogh, kalmir, talpik nar,kalmuru	Leaves	Fresh organ	Pain
**72.**	*Grewia tenax* (Forssk.) Fiori	Tiliaceae	Pootooroo, pootroo	Stem	Decoction	Cough, flank pain
**73.**	*Hammada saliocornica* (Moq.) Lijin.	Chenopodiaceae	Zaaz, jar, terat, peshker, rems, jaru	Leaves	Fresh organ decoction	Antipyretic, sensitivity of bees bite, wound healing
**74.**	*Heliotropium bacciferum* Forssk.	Boraginaceae	Aftabparast, ramram,defrak, rafetork, debrak, mispara	Aerial parts	Fresh organ	Wound bleeding, wound healing
**75.**	*Heliotropium europaeum* L.	Boraginaceae	Kolohmu, balghandu	Leaves, flower, seed, twigs	Brew fresh organ	Gout, cardiac tonic, headache, kidney stone, worm repellent
**76.**	*Herniaria hirsuta* L.	Paranychiaceae	Alafe fatgh kork alud	Aerial parts	Decoction brew	Washing wound & eye, kidney stone, almost all of kidney & bladder diseases, jaundice, female secretion
**77.**	*Hippocrepis unisilliquosa* L.	Papilionaceae	Naal asbi	Aerial parts	Fresh organ	Wound healing
**78.**	*Hymenocarpus circinnatus* (L.) Savi	Papilionaceae	-	Aerial parts	Fresh organ	Abscess
**79.**	*Hyoscyamus muticus* Bornm.	Solanaceae	Bazrolbanj	Seed	Smokes fumes	Toothache
**80.**	*Juniperus excelsa* M.B.	Cupressaceae	Ouras, abras, aras, hooras, gazkooh	Leaves, fruit	Decoction fresh organ	Rheumatism, dermal allergies, joint pain, back pain, foot pain, earache, diarrhea
**81.**	*Lagoecia cuminoides* L.	Umbelliferae	-	Aerial parts	Fresh organ	Bile stone repellent
**82.**	*Lallemantia royleana* Benth.	Lamiaceae	Balangu	Seed	Powder	Gum bleeding, psychotic disease, tonic
**83.**	*Launaea nudicaulis *(L.) Hook. f.	Compositae	Kahusa	Leaves	Fresh organ	Fever in children
**84.**	*Launaea procumbens* (Roxb.)	Compositae	Bonmoghi , nonak	Leaves	Fresh organ	Urination difficulty in children
**85.**	*Lavandula stricta* Del.	Lamiaceae	Ostokhodoos, ghadaar	Aerial parts	Fresh organ decoction	Rheumatism, cold, bone pain, carminative, abdominal cramps
**86.**	*Leptadenia pyrotechnica* (Forssk.)	Asclepiadaceae	Shahm nar, shahm oshtori, garishahk	Aerial parts	Powder	Carminative, wart, cutaneous fungal disease
**87.**	*Lycium Shawii* Roemer & Schult	Solanaceae	Dehir, zirok, dish	Twigs, leaves, fruit	Decoction	Gastric ailments, wound healing
**88.**	*Malva parviflora* L.	Malvaceae	Zazagh, sholaki	Seed	Decoction	Cold
**89.**	*Mentha longifolia * (L.) Hudson	Lamiaceae	Poden, pishe	Leaves, root	Decoction fresh organ brew	Carminative, diarrhea, cold, gastric ailments, stomachache, headache, antipyretic
**90.**	*Mentha mozaffariani* Jamzad	Lamiaceae	Poden kuhi	Leaves, twigs	Brew fresh organ	Cooling, diarrhea, stomachache, headache, carminative
**91.**	*Mesembryanthemum nodiflorum *L.	Aizoaceae	Hooshalang, ria, kheizaran,	Aerial parts	Decoction	Hives
**92.**	*Micromeria persica* Boiss.	Lamiaceae	Ovshen estaku	Leaves	Decoction	Acute fever, cold, stomachache, bone pain, carminative, abdominal discomforts
**93.**	*Myriophyllum verticallatum* L.	Haloragaceae	Partavoosi	Leaves	Fresh organ	Antipyretic, chronic dysentery, children cold
**94.**	*Nannorhops Ritchieana* H. Wendl.	Palmae	Daaz	Young leaves	Fresh organ	Diarrhea
**95.**	*Nerium indicum* Miller	Apocynaceae	Gish, kharzahreh	Leaves, latex	Fresh organ	Joint pain, gonalgia, foot pain, foot & hand edema remedy
**96.**	*Ochradenus Aucheri* Boiss.	Resedaceae	Shahm	Twigs, fruits	Brew decoction fresh organ	Stomachache, neck pain, pectoralgia
**97.**	*Otostegia Aucheri* Boiss.	Lamiaceae	Mesvake joojeh tighi	Root	Brew decoction fresh organ	Hair tonic, strengthening gums, dental cleaning & brightness, prevention of hair loss
**98.**	*Otostegia persica* (Burm.) Boiss.	Lamiaceae	Golgoder, gol khari, khoransh, golder	Leaves, flower, thistle	Brew decoction fresh organ	Cardiac distress, reducing palpitation, regulating blood pressure, laxative, carminative, antipyretic, cough, headache, gastric discomfort, parasite repellent
**99.**	*Pentatropis spiralis* (Forssk.) Decne.	Asclepiadaceae	Shahm	Roots	Decoction	Astringent, tonic, cooling, gonorrhea
**100.**	*Pergularia tomentosa* L.	Asclepiadaceae	Keshtu,helayah, labashir	Leaves	Powder fresh organ	Remedy for wounds in scorpions bite, wound healing
**101.**	*Perovskia artemisioides* Boiss.	Lamiaceae	-	Seed	Fresh organ	Rash, bone pain
**102.**	*Phragmithes australis* (Cav.) Trin. Ex Steud.	Gramineae	Ney, ghalam	Root	Brew	parasitic disease of stomach & intestine, flatulency
**103.**	*Physalis divaricata* D. Don.	Solanaceae	Kank	Leaves	Fresh organ	Abdominal pain in children
**104.**	*Pistacia atlantica* Desf.	Anacardiaceae	Sogand, bane, gan	Leaves, flower, resin	Decoction fresh organ powder	Acne, diarrhea, septic sore throat, back pain, old wounds, expectorant, infant GI tonic, children flatulency, anti rash, chest pain
**105.**	*Plantago amplexicaulis* Cav.	Plantaginaceae	Sialdaneh, danich, lajane, spiosh	Aerial parts	Powder Decoction syrup	Diarrhea, chest pain, strengthening children skeleton, stomachache, heatstroke, wounds, edema repellent
**106.**	*Platychaete glaucescense* (Boiss.) Boiss.	Compositae	Khormakharoo,mangoru, mangolo, kaskekharu	Leaves	Powder decoction	Wound healing, stomachache
**107.**	*Prosopis cineraria* (L.) Durce	Mimosaceae	Kahoor	Leaves, flower, resin	Fresh organ powder	Cutaneous fungal disease, wound healing, anemia in pregnant women, diarrhea
**108.**	*Pteropyrum Aucheri* Jaub. & Spach	Polygonaceae	Parand, patant, ostaparang, sidaf	Leaves, flower, root, stem	Decoction fresh organ	Bone, hand, leg & Knee pain, toothache, headache, back pain, wound healing, washing wounds
**109.**	*Pycnocycla Aucherana* Decne. Ex Boiss.	Umbelliferae	Sagdandan	Leaves, stem	Fresh organ	Back, leg & other part muscles pain
**110.**	*Ranunculus muricatus* L.	Ranunculaceae	Alale	Aerial parts	Fresh organ	Antipyretic
**111.**	*Reseda Aucheri* Boiss.	Resedaceae	Domroobahi, roogardesh, roozgardesh,gonavak, gararuz, dun eshtoru, gadukh	Leaves	Fresh organ	Remove the toxicity & sensitivity of snake bite, insect bite, scorpions bite
**112.**	*Rhazya stricta* Decne.	Apocynaceae	Eshvarak, kheshbarg, isur	Leaves	Decoction fresh organ	Bone pain, rheumatism, joint pain, toothache, eye pain
**113.**	*Rhizophora mucronata* Poir.	Rhizophoraceae	Chandal, chantela	Stem bark	Powder	Wound healing
**114.**	*Rumex dentatus* L.	Polygonaceae	Naazdolat	Seed	Decoction	Menstruation regulator, stops bleeding during menstruation
**115.**	*Salvadora persica* L.	Salvadoraceae	Chooch, chooj, raak, derakhte mesvak	Leaves, root	Toothpick	Headache, joint pain, cleaning teeth, strengthening gum
**116.**	*Salvia macrosiphon* Boiss.	Lamiaceae	Buing	Seed	Powder	Weakness, regulating cardiac action during pregnancy, lethargy after child birth
**117.**	*Salvia Mirzayanii *Rech. F. & Esfand.	Lamiaceae	Moortalkh, marve tahl, shir ghanam, mor porzu	Leaves	Powder decoction	Heart burn, diarrhea, emesis, stomachache, abdominal pain, flatulency, cooling, hyperlipidemia, hyperglycemia, jaundice, joint pain, headache, wound healing, scorpions scurry
**118.**	*Salvia Sharifii *Rech. F. & Esfand.	Lamiaceae	Borzoi, borooj, babriz, marmareshk	Seeds	Powder Decoction syrup	Emollient, cooling, wounds, diarrhea
**119.**	*Samolus Valerandi* L.	Primulaceae	Alaf juibari	All parts	Decoction	Astringent
**120.**	*Scorzonera paradoxa *Fisch. & C.A. Mey.	Compositae	komboluh	Bulb	Fresh organ	Laxative
**121.**	*Solanum incanum* L.	Solanaceae	Limoo aboojahl, limoo torgi, genj torgi, gelgelengak tourgi	Fruit, seed	Decoction	Wound, blotch, pustule treatment
**122.**	*Sonchus asper* (L.) Hill	Compositae	Shirtighak	Leaves, root, stem, flower, fruit	Fresh organ	Earache, asthma, chest discomforts, organ inflammation
**123.**	*Sophora mollis* (Royle) Backer	Papilionaceae	Talkhak, kalkhak	Root, seed, leaves	Decoction poultice	Cholera, irregular bile secretion, laxative, cathartic
**124.**	*Stachys inflata *Benth	Lamiaceae	Mohrkhari	Leaves	Decoction powder	Stomachache
**125.**	*Suaeda fruticosa* (L.)	Chenopodiaceae	Jar, kakol masilela	Leaves	Decoction	Jaundice
**126.**	*Tamarix dioica* Roth.	Tamaricaceae	Gaz	Stem bark, gall leafy branches	Poultice	Astringent, diarrhea, dysentery, cough, wound
**127.**	*Tamarix masqatensis* Bge.	Tamaricaceae	Gaz, gaze roodkhaneh, gazak	Leaves	Decoction	Joint pain, bone pain, softening muscle
**128.**	*Tanacetum fruticulosum* Ledeb.	Compositae	Dermene shah	Leaves	Fresh organ	Stomachache, abdominal pain, flatulency
**129.**	*Taverniera spartea* (Burm, f.) DC.	Papilionaceae	Laati, horosh nar	Stem	Decoction	Bone fractures
**130.**	*Tecomella undulate *(Roxb.) G. Don.	Bignoniaceae	Anare sheytani, anare aboojahl	Whole plant	Fresh organ	liver and gastrointestinal diseases
**131.**	*Tephrosia persica* Boiss.	Papilionaceae	Madkinak, bolbolengu	Leaves	Fresh organ	Scorpions bite, snake bite treatment
**132.**	*Teucrium orientale* (L.)	Lamiaceae	Golmaash	Leaves, flower	Decoction	Hoarseness
**133.**	*Teucrium pollium* L.	Lamiaceae	Kerishk, kalpuru	Flower, leaves, seed	Powder decoction Fresh organ	Stomachache, abdominal pain, flatulency, diarrhea, regulating blood pressure, menstruation in newly born ladies, measles, eye pain, headache, scorpions bite, snake bite, wound healing
**134.**	*Teucrium stocksianum* Boiss.	Lamiaceae	Kalpure kuhi, krishk daii	Leaves	Decoction Powder Fresh organ	Stomachache, abdominal pain, flatulency, toxicity, emesis, stomach acidification, regulating blood pressure, lipid lowering, newly born ladies recovery
**135.**	*Trianthema portulacastrum* L.	Aizoaceae	Vizakh, gooshe gorbeh	Whole plant	Powder	Cathartic, laxative, anemia, hemorrhoid, polydipsia, inflammation, pain relief, stomach tonic
**136.**	*Tribulus macropterus *Boiss.	Zygophyllaceae	Naalook	Leaves, flower, fruit	Decoction	Kidney pain & discomfort
**137.**	*Tribulus terresteris* L.	Zygophyllaceae	Kharkhasak, naalook, golezarde khaari	Leaves	Decoction	Kidney pain
**138.**	*Trichodesma africanum* (L.) R. Br.	Boraginaceae	Chaarmaahang	Root, leaves	Brew decoction	Cold, tightening bone fracture, abdominal pain, mouth ulcers, measles, scarlet fever, chickenpox, headache, emollient, chest congestion, children constipation
**139.**	*Vitex agnus-castus* L.	Verbenaceae	Bangela	Leaves, fruit, flowering twigs	Brew decoction	Cold, carminative, energizer, sedative, anticonvulsant, reducing libido
**140.**	*Vitex Negundo* L.	Verbenaceae	Bangela	Leaves, root, stem bark	Fresh organ	Toothache, rheumatism, carminative, anti-worm
**141.**	*Vitex trifolia *L.	Verbenaceae	Felfel khari, bamplusakh	Seed	Fresh organ	Ant repellent
**142.**	*Withania coagulans *(Stocks) Dun.	Solanaceae	Kheshtbargekasergkani	Fruit, seed	Fresh organ	Sedative, diuretic, dyspepsia, flatulency, intestine disorders, emetic, antidote
**143.**	*Withania somnifera* (L.) Dun.	Solanaceae	Mayepanir	Fruit	Fresh organ	Migraine, digestive disorders, hypnotic
**144.**	*Zataria multiflora* Boiss.	Lamiaceae	Oshen, azgand	Leaves	Powder Brew Fresh organ	Cold, diarrhea, stomachache, carminative, chest pain, headache, toothache, wound healing, fatigue, antipyretic, bone pain, earache, measles, reducing blood lipid & glucose
**145.**	*Zhumeria Majdae* Rech. F. & Wendelbo	Lamiaceae	Moorkhash, marvkhash, moorkhosh	Leaves	Powder Decoction fresh organ	Stomachache
**146.**	*Ziziphora tenuir* L.	Lamiaceae	Kakooti, golmoshkoo, ostokhodus, mongorush, hard angoshh	Aerial parts	Brew decoction	Gastric discomfort, cold, fever, diarrhea
**147.**	*Ziziphus jujuba* Mill.	Rhamnaceae	Annab	Fresh fruit, dried fruit	Decoction	Laxative, sedative, diuretic, emollient, chest disease
**148.**	*Ziziphus nummularia* (Burm. F.) Wight & Arn.	Rhamnaceae	Ramalik	Leaves, fruit	Decoction Fresh organ	Acne, sore throat, bleeding gums, joint pain, appetizing, gastric tonic
**149.**	*Zygophyllum qatarense* Hadidi	Zygophyllaceae	Shirmerku	Leaves, twigs	Powder Fresh organ	Wound healing, earache
**150.**	*Zygophyllum simplex* L.	Zygophyllaceae	Ghich	Seed, leaves	Brew	Eye disorders, worm killing

## Discussion

There are good network and several phytopharmaceutical industries and a technical wealth of botanical and herbal medicine experts available in Iran, however there has been little effort to document the volume and impact of medicinal plants in this country. More successful efforts are in progress about these fields. The Traditional Medicine Chancellery in the Iranian Ministry of Health and Medical Education was established in 2012 and for the first time in Iranian medical history, offering the postgraduate PhD degrees in Persian traditional medicine and traditional pharmacy sciences in Iranian universities of medical sciences were started from six years ago. Hopes to find more achievements especially in the ethnobotany and ethnomedicine disciplines are flourishing.

By doing this ethnobotanical research and after discussions with the people of Hormozgan province it was learnt that they are very close to the nature like other parts of Iran and the plants listed in the Table 1 are very much used by them for the variety of ailments. The recorded information revealed that the painful ailments, gastrointestinal, and dermatological disorders are in the top list of diseases that are treated by native plants. Plant specimens were belonging to 53 families and the most representative families were Lamiaceae and Compositae with 18 and 17 species, respectively, followed by Papilionaceae, Solanaceae, Asclepiadaceae and Umbelliferae, each with less than ten species. 

The ethnobotanical usage of medicinal plants in this is interesting and monopolizing and leads researchers and other medical and pharmaceutical experts to investigate further ethnopharmacological and pharmacognostical investigations (Attar et al., 2004 ▶; Soltanipoor, 2005 ▶; Soltanipoor, 2006 ▶). In this way, some species may be used in herbal drug preparation after the confirmation of their therapeutic efficacy and extraction of their active natural ingredients. Although the indigenous knowledge about plants is very important and useful, clinical trials and pharmacological studies should be done to prove their definite phytotherapical effects (Kazemi et al., 2012 ▶; Kunwar et al., 2010 ▶; Ghassemi Dehkordi et al., 2012 ▶; Zolfaghari et al., 2012 ▶).

During this survey, we completed and compared traditional and folk medicines information using phytotherapeutical and medicinal plants books and literature (Amin, 1991 ▶; BHP, 1983; Boger et al., 2006 ▶; Emami et al., 2010 ▶; IHP, 2002 ▶; PDR, 2000 ▶; WHO, 2007 ▶). The uses of several of these plants are consistent with our latest pharmacognostical and pharmacological findings (Asadipour et al., 2003 ▶; Ghannadi et al., 2000 ▶; Ghannadi et al., 2010 ▶; Ghannadi and Davoodi, 2012a ▶; Ghannadi et al., 2012b ▶; Jaffary et al., 2000 ▶; Jaffary et al., 2004 ▶; Minaiyan et al., 2005 ▶; Mohagheghzadeh et al., 2000a ▶; Mohagheghzadeh et al., 2000b ▶; Mohagheghzadeh et al., 2004 ▶; Sadraei et al., 2003a ▶; Sadraei et al., 2003b ▶; Shams Ardekani et al., 2005 ▶; Soltanipoor et al., 2003 ▶).

There has been relatively little basic research on the plants of Hormozgan province, Iran. This paper indicates that indigenous herbal knowledge is still alive in Iran and local people of Hormozgan province tend to use medicinal herbs and natural health products of their ecosystems for primary healthcare needs. 

The ethnobotanical survey of Hormozgan province allowed us to document the persistency of a number of traditional uses of medicinal plants, most of them are unique and original and potentially interesting as a basis for future research works.
